# Characterization of 65 Epitope-Specific Dystrophin Monoclonal Antibodies in Canine and Murine Models of Duchenne Muscular Dystrophy by Immunostaining and Western Blot

**DOI:** 10.1371/journal.pone.0088280

**Published:** 2014-02-07

**Authors:** Kasun Kodippili, Lauren Vince, Jin-Hong Shin, Yongping Yue, Glenn E. Morris, Mark A. McIntosh, Dongsheng Duan

**Affiliations:** 1 Department of Molecular Microbiology and Immunology, University of Missouri, Columbia, Missouri, United States of America; 2 Wolfson Centre for Inherited Neuromuscular Disease, RJAH Orthopaedic Hospital, Oswestry, and Keele University, Keele, Staffordshire, United Kingdom; Medical College of Georgia, United States of America

## Abstract

Epitope-specific monoclonal antibodies can provide unique insights for studying cellular proteins. Dystrophin is one of the largest cytoskeleton proteins encoded by 79 exons. The absence of dystrophin results in Duchenne muscular dystrophy (DMD). Over the last two decades, dozens of exon-specific human dystrophin monoclonal antibodies have been developed and successfully used for DMD diagnosis. Unfortunately, the majority of these antibodies have not been thoroughly characterized in dystrophin-deficient dogs, an outstanding large animal model for translational research. To fill the gap, we performed a comprehensive study on 65 dystrophin monoclonal antibodies in normal and dystrophic dogs (heart and skeletal muscle) by immunofluorescence staining and western blot. For comparison, we also included striated muscles from normal BL10 and dystrophin-null mdx mice. Our analysis revealed distinctive species, tissue and assay-dependent recognition patterns of different antibodies. Importantly, we identified 15 antibodies that can consistently detect full-length canine dystrophin in both immunostaining and western blot. Our results will serve as an important reference for studying DMD in the canine model.

## Introduction

Duchenne muscular dystrophy (DMD) is an X-linked degenerative muscle disorder. It is caused by frame shift or frame interruption mutations of the dystrophin gene [1]. The 2.3 megabase dystrophin gene is one of the largest known genes representing roughly 0.1% of the genome [2]. The dystrophin gene contains 79 exons and it translates into a 427 kD cytoskeletal protein [3], [4]. Dystrophin is predominantly expressed in skeletal and cardiac muscles [5]. It belongs to the β-spectrin/α-actinin protein family [6]. Dystrophin has four structurally distinctive domains. The first 240 amino acid residues form the actin-binding N-terminal domain. Next is a long rod-shaped central domain containing 24 spectrin-like repeats and four proline-rich hinges. The third domain is the cysteine-rich domain. The last 420 amino acid residues constitute the C-terminal domain [7]. Dystrophin localizes to the cytoplasmic surface of the sarcolemma in striated muscles [8]. It establishes a mechanical link between the extracellular matrix and the actin cytoskeleton (reviewed in [9], [10]).

Dystrophin-specific antibodies have played a pivotal role in the discovery and subsequent characterization of the dystrophin protein [4], [8], [11]. These antibodies have also been used as a tool for differential diagnosis of various types of muscular dystrophy [12]–[14]. In light of research and clinical needs, Morris and colleagues developed a series of epitope-specific dystrophin monoclonal antibodies (reviewed in [15]). These antibodies recognize unique epitope(s) in different exon(s) and thus can be used to precisely map gene deletion at the protein level [16], [17]. Besides the diagnostic value, these antibodies have also been widely used to study revertant fibers and smaller non-muscle isoforms of dystrophins [18]–[21].

Epitope-specific dystrophin monoclonal antibodies were initially generated to react with human dystrophin [22]. Interestingly, some of these antibodies also cross-reacted with dystrophins in other species. This provides an excellent opportunity for applying human dystrophin antibodies in preclinical animal studies. Dystrophin-deficient dogs are genetically and clinically comparable to human patients. Experimental therapies performed in dystrophic dogs are expected to more accurately predict the outcome of human trials [23]. To better characterize preclinical study in the canine model, we evaluated 65 dystrophin monoclonal antibodies in the heart and skeletal muscle of normal and dystrophic dogs by immunostaining and western blot. Since these antibodies have not been systemically analyzed in mice either, we also included striated muscles from wild type C57Bl/10 (BL10) and dystrophin-deficient mdx mice in the study.

## Materials and Methods

### Experimental Animals

All animal experiments were approved by the institutional animal care and use committee of the University of Missouri and were in accordance with NIH guidelines. Experimental dogs were produced in house by artificial insemination using semen from affected golden retriever, Corgi and Labrador dogs [23]–[25]. Diagnosis was made by PCR genotyping using umbilical cord and confirmed by elevated creatine kinase levels [24], [25]. Experimental dog tissues (from two normal and two affected dogs) were obtained at necropsy from adult dogs that were euthanized for other studies [24], [26], [27]. Specifically, the cranial tibialis muscle was used as the representative of skeletal muscle. The heart sample was from the posterior wall of the left ventricle. Experimental BL10 (C57BL/10ScSn) and mdx (C57BL/10ScSn-Dmd^mdx^/J) mice were obtained from The Jackson Laboratory (Bar Harbor, ME). Experimental mouse tissues (from two normal and two dystrophin-null mdx mice) were the anterior tibialis muscle and the whole heart.

### Monoclonal Antibodies

Sixty-five human dystrophin monoclonal antibodies were studied for their specificity and selectivity against mouse and dog dystrophin (Table S1). Five of these antibodies were purchased from commercial suppliers. Specifically, Dys-1 (clone Dy4/6D3, IgG2a), Dys-2 (clone Dy8/6C5, IgG1), Dys-3 (clone Dy10/12B2, IgG2a) and DysB (clone 34C5, IgG1kappa) were from Novacastra (Newcastle, UK). MANDYS8 (clone 8H11, IgG2b) was from Sigma-Aldrich (St Louis, MO). Remaining 60 antibodies were from the MDA Monoclonal Antibody Resource located at the Wolfson Centre for Inherited Neuromuscular Disease, RJAH Orthopaedic Hospital, Oswestry, UK (www.glennmorris.org.uk/mabs.htm). The detailed method of monoclonal antibody production has been reported before [22], [28], [29]. Briefly, a recombinant human dystrophin protein fragment was produced in E. coli and used to immunize a mouse. Hybridoma was then produced by the fusion of mouse myeloma cells and splenocytes.

### Immunofluorescence Staining

Freshly isolated muscle tissue was snap frozen in liquid nitrogen 2-methylbutane (isopentane) cryobath in optimal cutting temperature media (Sakura Finetek, Torrance, CA). The muscle tissue block was cut into 8 µm sections with a Richard-Allan HM 505 E microtome cryostat (Thermo Fisher, Kalamazoo, MI). Cryosections were air dried and then incubated with Fab-c (rabbit anti-mouse IgG) for one hour at room temperature [30]. After the slides were washed with phosphate buffered saline (PBS) three times (5 minutes each), they were blocked with 20% goat serum in PBS at room temperature for 30 minutes and washed again with PBS three times, for 5 minutes each. The muscle sections were then incubated with a human dystrophin monoclonal antibody (1∶100, diluted in 1% goat serum in PBS) at 4 °C overnight. The following day, the slides were washed with 1% goat serum in PBS three times, for 5 minutes each and then incubated with Alexa 594-conjugated goat anti-mouse secondary antibody (1∶100, diluted in 1% goat serum in PBS; Invitrogen-Molecular Probe, Carlsbad, CA) for 30 minutes at room temperature. The muscle sections were washed again with 1% goat serum in PBS three times, 5 minutes each time. Finally, slides were covered with a drop of Citifluor antifadent mounting medium (Electron Microscopy Sciences, Hatfield, PA) and observed under a Nikon Eclipse E800 fluorescence microscope (Nikon Instruments, Melville, NY). Photomicrographs were taken with a Qimage REtiga 1300 camera (QImaging Corporate, Surrey, BC, Canada) at a fixed condition for all slides. Staining intensity was ranked as negative (−), faint (barely detectable), weak positive (+), positive (++) and strong positive (+++)(Figure S1). Immunostaining was performed once to screen all 65 antibodies, while those that did not produce very clear results were repeated. Antibodies that gave species and tissue specific results and those that did not detect canine or murine dystrophin at all were tested in triplicate to verify initial observations.

### Western Blot

Freshly dissected muscle tissue was snap frozen in liquid nitrogen. Muscle was then homogenized using liquid nitrogen-cooled mortar and pestle in a homogenization buffer containing 10% sodium dodecyl sulfate (SDS), 5 mM ethylenediaminetetraacetic acid, 62.5 mM Tris-HCl (pH 6.8) and 2% protease inhibitor (Roche, Indianapolis, IN). Homogenate was spun at 14,000 rpm for 2 minutes (Eppendorf centrifuge, model 5417C; Eppendorf-Netheler-Hinz GmbH, Hamburg, Germany). The supernatant was used for western blot. Protein concentration was determined using the Bio-Rad DC protein assay kit (Bio-Rad, Hercules, CA). 30 µg of protein were loaded on a 3% stacking/6%separating SDS-polyacrylamide gel and run for 3.5 hours at 100 V. Following electrophoresis, protein was transferred to a polyvinylidene fluoride (PVDF) membrane. The PVDF membrane was blocked with 5% milk in Tris-buffered saline (TBS)-Tween (TBST) solution (containing 1x TBS and 0.1% Tween 20) for one hour at room temperature. Subsequently the PVDF membrane was incubated with a human dystrophin monoclonal antibody (1∶100 dilution in 5% milk/TBST overnight at 4°C). The membrane was washed in TBST for 5 minutes and then incubated with the horseradish peroxidase conjugated goat anti-mouse IgG secondary antibody (1∶2,000 dilution in TBST, Santa Cruz, Dallas, TX) for one hour at room temperature. After a final wash with TBST for 5 minute, signals were detected using the ECL system (GE Healthcare Biosciences, Pittsburgh, PA). Protein loading was confirmed with Ponceau S staining. The intensity of the western blot band was ranked as negative (−), weak positive (weak) and positive (pos) (Figure S1). Western blots were performed once to screen all 65 antibodies, while those that did not produce very clear results were repeated. Antibodies that gave species and tissue specific results and those that did not detect canine or murine dystrophin at all were tested in triplicate to verify initial observations.

## Results

### Antibodies Reacting with both Canine and Murine Full-length Dystrophin

To streamline the comparison, standardized protocols were used throughout the study. Immunofluorescence staining was graded as strongly positive (+++), positive (++), weak positive (+), faint (barely detectable) and negative (−) (Figure S1). Western blot was graded as positive (pos), weak positive (weak), and negative (−) (Figure S1).

**Figure 1:**
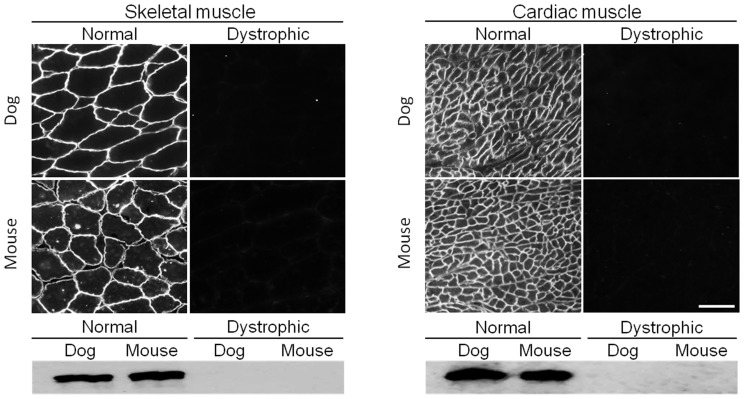
A representative example of antibodies that react with both canine and murine full-length dystrophin. Immunofluorescence staining and western blot were performed on mouse and dog muscles from normal and dystrophic animals using MANDYS1, an epitope specific antibody against exons encoding repeats 10 and 11 of the central rod domain of dystrophin. Scale bar, 50 µm.

Among 65 human antibodies, 13 antibodies reacted with both canine and mouse dystrophin on immunofluorescence staining (Figure 1, Table 1). On western blot, these antibodies also successfully detected the full-length 427 kD dystrophin band in normal muscle (Figure 1, Table 1). MANDYS1 (against repeats 10 and 11 of the central rod domain) is a good example of this group of antibodies (Figure 1). It produced clear and intense sarcolemmal staining under the microscope and it also detected the full-length dystrophin protein on western blots (Figure 1). Interestingly, MANDYS1 also revealed a weak 100 kD band on all heart western blots irrespective of the species (mouse or dog) and disease status (with or without muscular dystrophy) (Table 2). Similarly, several other antibodies from this group detected smaller cross-reactive bands on western blot. Specifically, MANDRA9 (against the C-terminal domain) showed a similar western blot pattern as that of MANDYS1 except that the cross-reactive band migrated to ∼230 kD (instead of 100 kD) on heart western blot (Table 2). MANEX44A (against repeat 17) consistently recognized a 200 kD band in every western blot independent of the source of the muscle tissue (normal or dystrophic, dog or mouse, heart or skeletal muscle) (Table 2). Finally, MANDRA4 (against the C-terminal domain) selectively reacted with two smaller bands (140 kD and 250 kD) in normal and dystrophic dog skeletal muscle western blot.

**Table 1:** A summary of selective antibodies based on their reactivity.

Antibody name	Ig subtype	Epitope specificity	Domain Specificity	Normal dog tissue				Dystrophic dog tissue				Normal mouse tissue				Dystrophic mouse tissue			
				Skeletal muscle		Cardiac muscle		Skeletal muscle		Cardiac muscle		Skeletal muscle		Cardiac muscle		Skeletal muscle		Cardiac muscle	
				IF	WB	IF	WB	IF	WB	IF	WB	IF	WB	IF	WB	IF	WB	IF	WB
***Antibodies that react with both canine and murine full-length dystrophin (MANDYS1 is illustrated in *** ***Figure 1*** ***)***																			
DysB	G1	Exon 10–12	H1-R2	+++	Pos	+++	Pos	_	_	_	_	++	Weak	++	Weak	_	_	_	_
MANDYS17	G1	Exon 26/27	R8	+	Pos	+	Pos	_	_	_	_	+	Pos	+	Pos	_	_	_	_
**MANDYS1**	**G2a**	**Exon 31/32**	**R10–R11**	**++**	**Pos**	**++**	**Pos**	**_**	**_**	**_**	**_**	**++**	**Pos**	**++**	**Pos**	**_**	**_**	**_**	**_**
MANDYS8	G2b	Exon 32	R11	+++	Pos	+++	Pos	_	_	_	_	+++	Pos	+++	Pos	_	_	_	_
MANEX44A	n.d.	Exon 44	R17	+++	Pos	+++	Pos	_	_	_	_	++	Pos	+++	Pos	_	_	_	_
MANEX46A	G1	Exon 46	R18	++	Pos	++	Pos	_	_	_	_	+	Pos	+	Weak	_	_	_	_
MANDRA9	G1	Exon 70–79	CT	+++	Pos	++	Pos	_	_	_	_	++	Pos	++	Pos	_	_	_	_
MANDRA2	G1	Exon 70–79	CT	++	Pos	+	Pos	_	_	_	_	+	Pos	+	Pos	_	_	_	_
MANEX7374A	G1	Exon 73/74	CT	++	Pos	++	Pos	_	_	_	_	++	Pos	++	Pos	_	_	_	_
MANDRA17	G1	Exon 74/75	CT	+++	Pos	++	Pos	_	_	_	_	+	Pos	++	Pos	_	_	_	_
MANDRA6	G1	Exon 75	CT	+++	Pos	++	Pos	_	_	_	_	+	Weak	+	Pos	_	_	_	_
MANDRA4	G1	Exon 77	CT	++	Pos	++	Pos	_	_	_	_	++	Pos	++	Weak	_	_	_	_
Dys-2	G1	Exon 77–79	CT	+	Weak	++	Pos	_	_	_	_	++	Pos	++	Pos	_	_	_	_
***Antibodies that only react with mouse dystrophin (MANEX1216B is illustrated in *** ***Figure 2*** ***)***																			
**MANEX1216B**	**G2a**	**Exon 12–16**	**R1–R3**	**_**	**_**	**_**	**Weak**	**_**	**_**	**_**	**_**	**+**	**Pos**	**+**	**Pos**	**_**	**_**	**_**	**_**
MANEX45B	G1	Exon 45	R17	_	_	_	Weak	_	_	_	_	+	Weak	+	Pos	_	_	_	_
***MANEX4850D only reacts with dog dystrophin (see details in *** ***Figure 3*** ***)***																			
MANEX4850D	G2b	Exon 48–50	R19-H3	++	Pos	++	Pos	_	_	_	_	_	_	_	_	_	_	_	_
***Antibodies that only only work for immunostaining (MANDYS111 is illustrated in *** ***Figure 4*** ***)***																			
**MANDYS111**	**G2a**	**Exon 40/41**	**R15**	**+++**	**_**	**+++**	**_**	**_**	**_**	**_**	**_**	**++**	**_**	**++**	**_**	**_**	**_**	**_**	**_**
MANDYS105	G1	Exon 37–46	R13–R18	+++	_	+++	Weak	_	_	_	_	+++	_	+++	_	_	_	_	_
MANDYS103	G2a	Exon 43	R16	++	_	++	_	_	_	_	_	+	_	+	_	_	_	_	_
MANDYS106	G2a	Exon 43	R16	++	_	++	_	_	_	_	_	+	_	_	_	_	_	_	_
***Antibodies that only only work for western blot (MANDYS141 is illustrated in *** ***Figure 5*** ***)***																			
**MANDYS141**	**G2b**	**Exon 38**	**R14**	**_**	**Pos**	**_**	**Pos**	**_**	**_**	**_**	**_**	**_**	**Pos**	**_**	**Pos**	**_**	**_**	**_**	**_**
MANDRA13	M	Exon 75	CT	_	Pos	_	Pos	_	_	_	_	_	Pos	_	Pos	_	_	_	_
MANDRA14	G1	Exon 75	CT	_	Pos	_	Pos	_	_	_	_	_	Pos	+	Pos	_	_	_	_
***MANHINGE4A primarily reacts with cardiac dystrophin (see details in *** ***Figure 6*** ***)***																			
MANHINGE4A	G1	Exon 62	H4	faint	_	++	Pos	_	_	_	_	_	_	++	Pos	_	_	_	_
***Antibodies that do not recognize murine and canine dystrophin (MANEX8A is illustrated in *** ***Figure 7*** ***)***																			
MANEX6	G1	Exon 6	NT	_	_	_	_	_	_	_	_	_	_	_	_	_	_	_	_
**MANEX8A**	**G1**	**Exon 8**	**NT-H1**	**_**	**_**	**_**	**_**	**_**	**_**	**_**	**_**	**_**	**_**	**_**	**_**	**_**	**_**	**_**	**_**
Dys-3	G2a	Exon 9–10	H1-R1	_	_	_	_	_	_	_	_	_	_	_	_	_	_	_	_
MANHINGE3B	G2a	Exon 51	H3-R20	_	_	_	_	_	_	_	_	_	_	_	_	_	_	_	_

IF, immunofluorescence staining; Ig, immunoglobulin; WB, western blot.

CT, C-terminal domain; H, hinge; NT, N-terminal domain; R, spectrin-like repeat.

+++, strongly positive in immunostaining; ++, positive in immunostaining; +, weakly positive in immunostaining; −, negative in immunostaining.

Pos, positive in western blot; Weak, weakly positive in western blot.

**Table 2:** Antibodies that show cross-reactive bands in western blot.

Antibody name	Ig subtype	Epitope specificity	Domain Specificity	Normal dog tissue				Dystrophic dog tissue				Normal mouse tissue				Dystrophic mouse tissue			
				Skeletal muscle		Cardiac muscle		Skeletal muscle		Cardiac muscle		Skeletal muscle		Cardiac muscle		Skeletal muscle		Cardiac muscle	
				FL Dys	Cross reactions	FL Dys	Cross reactions	FL Utro*	Cross reactions	FL Dys	Cross reactions	FL Dys	Cross reactions	FL Dys	Cross reactions	FL Utro*	Cross reactions	FL Dys	Cross reactions
MANEX1A	G2a	Exon 1	NT	_	_	Pos	_	_	_	_	_	_	_	Pos	250 (w)	_	_	_	_
MANHINGE3C	M	Exon 8	NT-H1	Pos	400, 200	Pos	300	Weak	430	_	_	_	_	Weak	_	_	_	_	_
DysB	G1	Exon 10–12	H1-R2	Pos	_	Pos	300	_	_	_	_	Weak	_	Weak	_	_	_	_	_
MANEX1216B	G2a	Exon 12–16	R1–R3	_	150 (w), 130, 120 (w)	Weak	_	_	130, 120 (w)	_	_	Pos	_	Pos	380 (w)	_	_	_	_
MANEX1216A	G2a	Exon 14	R2–R3	_	160, 140	Pos	400 (w)	_	_	_	_	_	_	Pos	_	_	_	_	_
MANHINGE2A	G1	Exon 17	H2	Weak	130 (w)	_	_	Weak	400 (w), 250, 130, 120 (w)	_	_	_	_	Weak	_	_	_	_	_
MANDYS19	G1	Exon 20/21	R4–R5	Pos	300, 130, 110	Pos	_	Pos	300, 150 (w), 130, 110	_	_	Pos	_	Pos	_	_	_	_	_
Dys-1	G2a	Exon 26–30	R8–R10	Pos	400 (w)	Pos	_	_	_	_	_	Pos	_	Pos	_	_	_	_	_
MANDYS16	G2b	Exon 27/28	R8–R9	Pos	90	_	_	_	130 (w)	_	_	Pos	_	_	_	_	_	_	_
MANDYS1	G2a	Exon 31/32	R10–R11	Pos	_	Pos	100 (w)	_	_	_	100 (w)	Pos	_	Pos	100 (w)	_	_	_	100 (w)
MANDYS141	G2b	Exon 38	R14	Pos	170 (w), 100	Pos	100	_	170 (w), 100	_	100	Pos	200, 100	Pos	170 (w), 100	_	200, 100	_	100
MANDYS110	G1	Exon 38/39	R14	_	130, 120 (w)	Pos	_	_	_	_	_	_	_	Pos	_	_	_	_	_
MANDYS101	G2b	Exon 40/41	R15	Pos	130	Pos	_	_	_	_	_	_	_	_	_	_	_	_	_
MANDYS107	G2b	Exon 40/41	R15	Pos	400	Pos	_	_	_	_	_	Weak	_	_	_	_	_	_	_
MANDYS111	G2a	Exon 40/41	R15	_	160, 140 (w)	_	_	_	400, 160, 140	_	_	_	_	_	_	Weak	_	_	_
MANDYS124	G1	Exon 40/41	R15	Pos	_	Pos	_	Pos	250, 160, 140 (w)	_	_	Pos	_	Pos	_	_	_	_	_
MANEX44A	n.d.	Exon 44	R17	Pos	200	Pos	200	_	200	_	200	Pos	200	Pos	200	_	200	_	200
MANEX4748A	G2b	Exon 47/48	R18–R19	_	130	_	_	_	_	_	_	_	_	_	_	_	_	_	_
MANEX4850B	G1	Exon 48–50	R19-H3	Pos	110 (w)	Pos	_	_	_	_	_	_	_	Weak	_	_	_	_	_
MANEX4850D	G2b	Exon 48–50	R19-H3	Pos	300 (w)	Pos	_	_	_	_	_	_	_	_	_	_	_	_	_
MANEX50	n.a.	Exon 50	R19-H3	Pos	350, 200, 175 (w)	Pos	350, 200, 175 (w)	Weak	_	_	_	Pos	_	Pos	_	_	_	_	_
MANHINGE3A	G1	Exon 51	H3-R20	_	120 (w)	_	_	_	_	_	_	_	_	_	_	_	_	_	_
MANHINGE3B	G2a	Exon 51	H3-R20	_	140 (w)	_	_	_	_	_	_	_	_	_	_	_	_	_	_
MANHINGE4A	G1	Exon 62	H4	_	_	Pos	400 (w)	_	_	_	_	_	_	Pos	400 (w)	_	_	_	_
MANDRA9	G1	Exon 70–79	CT	Pos	_	Pos	230	_	_	_	230	Pos	_	Pos	230	_	_	_	230
MANCHO18	G1	Exon 75	CT	Pos	200, 140, 120 (w)	Pos	_	Weak	250, 200, 140, 120 (w)	_	_	Pos	_	Pos	_	Weak	_	_	_
MANDRA5	G2a	Exon 76/77	CT	Pos	140	Pos	_	Weak	250, 140	_	_	Pos	_	Pos	_	_	_	_	_
MANDRA4	G1	Exon 77	CT	Pos	140 (w)	Pos	_	_	250 (w), 140	_	_	Pos	_	Weak	_	_	_	_	_

*We suspect that these are full-length utrophin bands. Future studies are needed to confirm their identity.

The size of cross-reactive bands is marked. The molecular weight unit is kD. Weak cross-reactive bands are indicated with (w).

Ig, immunoglobulin; FL Dys, full-length dystrophin.

CT, C-terminal domain; H, hinge; NT, N-terminal domain; R, spectrin-like repeat.

Pos, positive in western blot; Weak, weakly positive in western blot.

### Species-specific Antibodies

**Figure 2:**
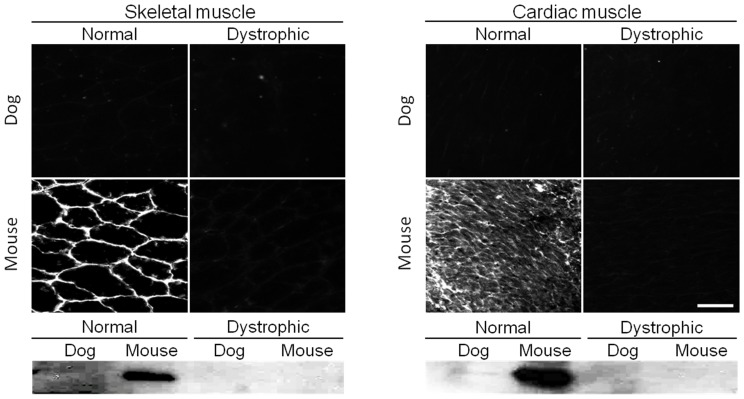
A representative example of antibodies that only react with mouse dystrophin. Immunofluorescence staining and western blot were performed on mouse and dog muscles from normal and dystrophic animals using MANEX1216B, an epitope specific antibody against exons encoding repeat 12 through 16 of the central rod domain of dystrophin. Scale bar, 50 µm.

All 65 antibodies described in this study are known to recognize human dystrophin (www.glennmorris.org.uk/mabs.htm). While the majority also reacted with murine and canine dystrophin, three antibodies displayed species-specificity. Two antibodies showed specificity to mouse muscle on immunostaining (Figure 2, Table 1). These are MANEX1216B (against R2–R3) and MANEX45B (against R17). Positive sarcolemmal staining was seen in normal mouse muscle only (Figure 2). However, western blot with these two antibodies yielded a more complex pattern. Both antibodies recognized full-length mouse dystrophin (heart and skeletal muscle). Interestingly, they also detected a very faint full-length band in normal dog heart (but not skeletal muscle) (Figure 2, Tables 1 and 2). In addition, MANEX1216B also yielded a few cross-reactive bands (120 to 150 kD) in dog skeletal muscle and a ∼380 kD weak cross-reactive band in normal mouse heart (Table 2).

**Figure 3:**
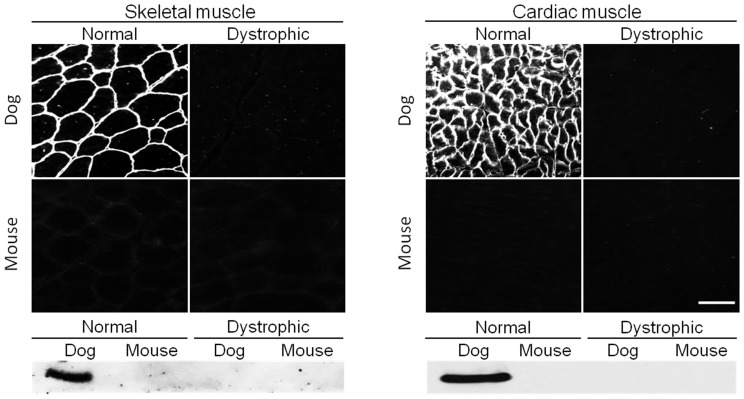
MANEX4850D only reacts with dog dystrophin. Immunofluorescence staining and western blot were performed on mouse and dog muscles from normal and dystrophic animals using MANEX4850D, an epitope specific antibody against exons encoding repeat 19 and hinge 3 of the central rod domain of dystrophin. Scale bar, 50 µm.

One antibody (MANEX4850D, against repeat 19 and hinge 3) recognized full-length canine, but not murine, dystrophin (Figure 3, Table 1). On immunostaining, MANEX4850D yielded strong sarcolemmal staining only in normal dog muscle (Figure 2). On western blot, MANEX4850D reacted with full-length dog dystrophin in both skeletal and cardiac muscle (Figure 3). Interestingly, it also revealed a weak ∼300 kD band in normal dog skeletal muscle (Table 2).

### Assay-specific Antibodies

**Figure 4:**
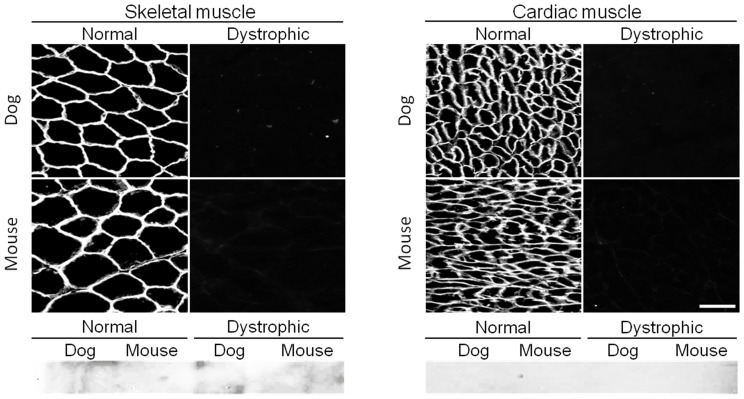
A representative example of antibodies that only work for immunostaining. Immunofluorescence staining and western blot were performed on mouse and dog muscles from normal and dystrophic animals using MANDYS111, an epitope specific antibody against exons encoding repeat 15 of the central rod domain of dystrophin. Scale bar, 50 µm.

Seven antibodies showed assay specificity. MANDYS111 (against R15), MANDYS105 (against R13–18), MANDYS103 (against R16) and MANDSY106 (against R16) essentially did not work on western blot (except for a weak band in dog heart western detected by MANDYS105). However, these four antibodies were able to light up the sarcolemma on immunofluorescence staining in nearly all settings (except for MANDSY106 which failed in mouse heart immunostaining) (Figure 4, Table 1).

**Figure 5:**
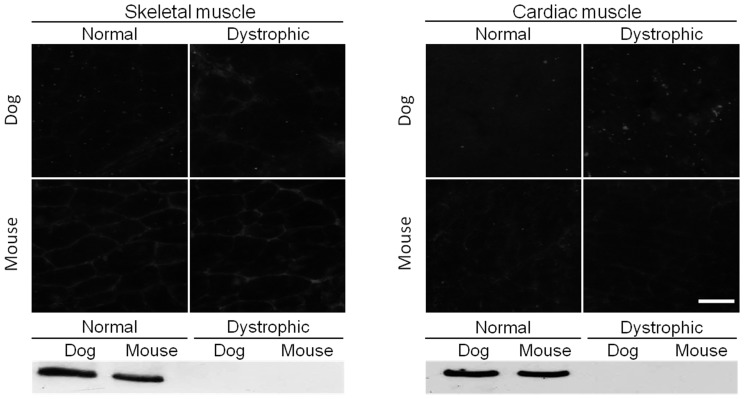
A representative example of antibodies that only work in western blot. Immunofluorescence staining and western blot were performed on mouse and dog muscles from normal and dystrophic animals using MANDYS141, an epitope specific antibody against exons encoding repeat 14 of the central rod domain of dystrophin. Scale bar, 50 µm.

On the other hand, MANDYS141 (against R14), MANDRA13 (against the C-terminal domain) and MANDRA14 (against the C-terminal domain) mainly worked in western blot but not immunostaining (except for MANDRA14 which yielded a weak positive signal in the mouse heart) (Figure 5, Table 1). All three antibodies successfully revealed the full-length 427 kD dystrophin protein on immunoblot (Figure 5, Table 2). Nevertheless, only MANDRA13 and MANDRA14 yielded a clean blot without additional bands while MANDYS141 also recognized some 100 to 200 kD bands in both normal and dystrophic muscle tissues (Table 2).

### Tissue-specific Antibodies

**Figure 6:**
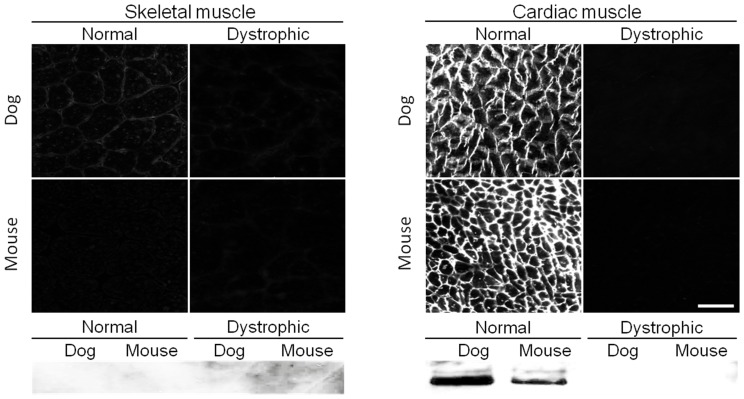
MANHINGE4A is a heart specific antibody. Immunofluorescence staining and western blot were performed on mouse and dog muscles from normal and dystrophic animals using MANDYS141, an epitope specific antibody against exons encoding hinge 4 of the central rod domain of dystrophin. Scale bar, 50 µm.

One antibody appeared to be tissue-specific. MANHINGE4A (against hinge 4) detected both mouse and canine dystrophin in the heart (Figure 6, Table 1). Besides a faint, barely detectable signal on normal dog skeletal muscle immunostaining, this antibody was not able to reveal any dystrophin from skeletal muscle (Figure 6, Table 1).

### Antibodies that Fail to React with Either Murine or Canine Dystrophin

**Figure 7:**
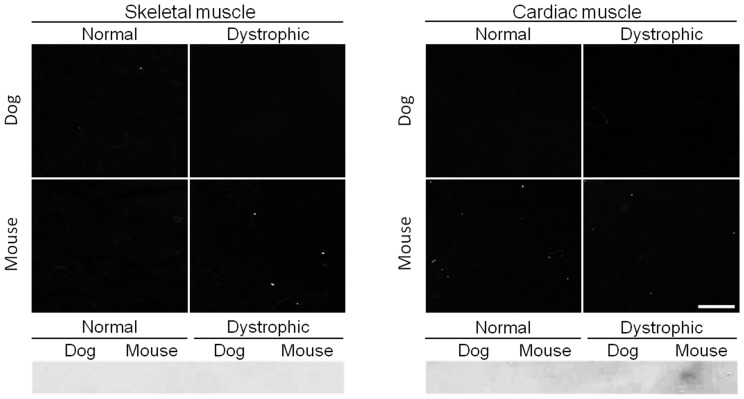
A representative example of antibodies that do not recognize murine and canine dystrophin. Immunofluorescence staining and western blot were performed on mouse and dog muscles from normal and dystrophic animals using MANEX8A. This antibody is developed to react with an epitope located in exons encoding the N-terminal domain and hinge 1. Scale bar, 50 µm.

Despite repetitive attempts, four antibodies (MANEX6, MANEX8A, Dys-3 and MANHINGE3B) did not show any reactivity against dystrophin from either mouse or canine tissue (Figure 7, Table 1). Nevertheless, it should be pointed out that these antibodies can recognize dystrophin in human tissues (www.glennmorris.org.uk/mabs.htm).

### Antibodies that Recognize a Band at the Size of Full-length Dystrophin in Dystrophic Muscle

There are 11 antibodies in this category (Table 3). Four antibodies (MANDYS19, MANDYS124, MANCHO11 and MANDRA16) revealed a definitive band while remaining seven antibodies only yielded a weak band (Table 3). Five antibodies (MANCHO11, MANDRA16 AND MANDRA3, MANCHO18 and MANDYS18) reacted with an approximately 400 kD band in dystrophin-deficient mouse and dog muscles. Among these five antibodies, three (MANCHO11, MANDRA16 and MANDRA3) showed the band in both skeletal muscle and heart western blot. One (MANCHO18) showed the band in skeletal muscle blot only and the other (MANDYS18) in heart blot only. Six antibodies (MANHINGE3C, MANHINGE2A, MANDYS19, MANDYS124, MANEX50 AND MANDRA5) detected a similar size band in dystrophic dog skeletal muscle only (Table 3). Intriguingly, these antibodies did not yield positive sarcolemmal labeling in immunostaining (Table 3, Table S1).

**Table 3:** Antibodies that revealed an approximately full-length size band in dystrophic muscle western blot.

Antibody name	Igsubtype	Epitope specificity	Domain Specificity	Dystrophic dog tissue				Dystrophic mouse tissue			
				Skeletal muscle		Cardiac muscle		Skeletal muscle		Cardiac muscle	
				IF	WB*	IF	WB*	IF	WB*	IF	WB*
MANHINGE3C	M	Exon 8	NT-H1	_	Weak	_	_	_	_	_	_
MANHINGE2A	G1	Exon 17	H2	_	Weak	_	_	_	_	_	_
MANDYS19	G1	Exon 20/21	R4–R5	_	Pos	_	_	_	_	_	_
MANDYS18	G2a	Exon 26	R8	_	_	_	Weak	_	_	_	Weak
MANDYS124	G1	Exon 40/41	R15	_	Pos	_	_	_	_	_	_
MANEX50	n.a.	Exon 50	R19-H3	_	Weak	_	_	_	_	_	_
MANCHO11	G1	Exon 72/73	CT	_	Pos	_	Pos	_	Pos	_	Pos
MANDRA16	G1	Exon 75	CT	_	Pos	_	Pos	_	Pos	_	Pos
MANDRA3	G1	Exon 75	CT	_	Weak	_	Weak	_	Weak	_	Weak
MANCHO18	G1	Exon 75	CT	_	Weak	_	_	_	Weak	_	_
MANDRA5	G2a	Exon 76/77	CT	_	Weak	_	_	_	_	_	_

*We suspect that these are full-length utrophin bands. Future studies are needed to confirm their identity.

IF, immunofluorescence staining; Ig, immunoglobulin; WB, western blot.

CT, C-terminal domain; H, hinge; NT, N-terminal domain; R, spectrin-like repeat.

−negative in immunostaining or western blot.

n.a., information not available.

Pos, positive in western blot; Weak, weakly positive in western blot.

### Antibodies Recommended for Canine Study

From 65 antibodies, we identified 15 as the most suitable antibodies for canine study (Table 4). These antibodies worked well for canine tissue on both western blot and immunostaining. They revealed expected sarcolemmal staining in tissue sections from normal dog heart and skeletal muscle. They also yielded one distinctive 427 kD band (without smaller cross-reactive bands) on western blot from normal dog muscle. Importantly, none of these antibodies reacted with muscle tissues from affected dogs (Table 4).

**Table 4:** Antibodies that we recommend for canine study (immunostaining and western blot).

Antibody name	Ig subtype	Epitope specificity	Domain Specificity	Normal dog tissue				Dystrophic dog tissue			
				Skeletal muscle		Cardiac muscle		Skeletal muscle		Cardiac muscle	
				IF	WB	IF	WB	IF	WB	IF	WB
MANHINGE1A	G1	Exon 8	NT-H1	+++	Pos	+	Pos	_	_	_	_
MANEX1011A	G1	Exon 10/11	H1-R1	+	Pos	+	Pos	_	_	_	_
DysB	G1	Exon 10–12	H1-R2	+++	Pos	+++	Pos	_	_	_	_
MANDYS17	G1	Exon 26/27	R8	+	Pos	+	Pos	_	_	_	_
MANDYS8	G2b	Exon 32	R11	+++	Pos	+++	Pos	_	_	_	_
MANDYS104	G2a	Exon 39–46	R14–R18	+++	Pos	++	Weak	_	_	_	_
MANEX44B	G5	Exon 44	R17	+	Pos	+	Pos	_	_	_	_
MANEX46A	G1	Exon 46	R18	++	Pos	++	Pos	_	_	_	_
MANEX47	G1	Exon 47	R18	++	Pos	+++	Pos	_	_	_	_
MANEX4850A	n.a.	Exon 48–50	R19-H3	+++	Pos	+++	Pos	_	_	_	_
MANDRA2	G1	Exon 70–79	CT	++	Pos	+	Pos	_	_	_	_
MANEX7374A	G1	Exon 73/74	CT	++	Pos	++	Pos	_	_	_	_
MANDRA17	G1	Exon 74/75	CT	+++	Pos	++	Pos	_	_	_	_
MANDRA6	G1	Exon 75	CT	+++	Pos	++	Pos	_	_	_	_
Dys-2	G1	Exon 77–79	CT	+	Weak	++	Pos	_	_	_	_

IF, immunofluorescence staining; Ig, immunoglobulin; WB, western blot.

CT, C-terminal domain; H, hinge; NT, N-terminal domain; R, spectrin-like repeat.

+++, strongly positive in immunostaining; ++, positive in immunostaining; +, weakly positive in immunostaining; −, negative in immunostaining.

Pos, positive in western blot; Weak, weakly positive in western blot.

Another set of antibodies was found as good candidates for dog muscle immunostaining only (Table 5). There are 15 antibodies in this group. These antibodies reacted with dog dystrophin on immunostaining and western blot. In addition, they also detected smaller cross-reactive bands in western blot (Table 5).

**Table 5:** Antibodies that we recommend for use in immunostaining only in canine study.

Antibody name	Ig subtype	Epitope specificity	Domain Specificity	Normal dog tissue						Dystrophic dog tissue					
				Skeletal muscle			Cardiac muscle			Skeletal muscle			Cardiac muscle		
				IF	WB		IF	WB		IF	WB		IF	WB	
					FL Dys	Cross reac.		FL Dys	Cross reac.		FL Dys	Cross reac.		FL Dys	Cross reac.
MANDYS19	G1	Exon 20/21	R4–R5	+++	Pos	300, 130, 110	+++	Pos	_	_	Pos	300, 150 (w), 130, 110	_	_	_
MANDYS18	G2a	Exon 26	R8	+	Pos	_	++	Pos	_	_	_	_	_	Weak	_
Dys-1	G2a	Exon 26-30	R8–R10	++	Pos	400 (w)	++	Pos	_	_	_	_	_	_	_
MANDYS1	G2a	Exon 31/32	R10–R11	++	Pos	_	++	Pos	100 (w)	_	_	_	_	_	_
MANDYS101	G2b	Exon 40/41	R15	+++	Pos	130	++	Pos	_	_	_	_	_	_	_
MANDYS107	G2b	Exon 40/41	R15	+++	Pos	400	+++	Pos	_	_	_	_	_	_	_
MANEX44A	n.d.	Exon 44	R17	+++	Pos	200	+++	Pos	200	_	_	_	_	_	_
MANEX4850B	G1	Exon 48–50	R19-H3	+++	Pos	110 (w)	+++	Pos	_	_	_	_	_	_	_
MANEX4850D	G2b	Exon 48–50	R19-H3	++	Pos	300 (w)	++	Pos	_	_	_	_	_	_	_
MANEX50	n.a.	Exon 50	R19-H3	+++	Pos	350, 200, 175 (w)	+++	Pos	350, 200, 175 (w)	_	Weak	_	_	_	_
MANDRA9	G1	Exon 70–79	CT	+++	Pos	_	++	Pos	230	_	_	_	_	_	_
MANCHO11	G1	Exon 72/73	CT	++	Pos	_	++	Pos	_	_	Pos	_	_	Pos	_
MANDRA16	G1	Exon 75	CT	+++	Pos	_	+++	Pos	_	_	Pos	_	_	Pos	_
MANDRA3	G1	Exon 75	CT	++	Pos	_	+	Pos	_	_	Pos	_	_	Weak	_
MANDRA4	G1	Exon 77	CT	++	Pos	140 (w)	++	Pos	_	_	_	_	_	_	_

Cross reac., cross-reactive band; FL Dys, full-length dystrophin; IF, immunofluorescence staining; Ig, immunoglobulin; WB, western blot.

CT, C-terminal domain; H, hinge; R, spectrin-like repeat.

+++, strongly positive in immunostaining; ++, positive in immunostaining; +, weakly positive in immunostaining; −, negative in immunostaining.

Pos, positive in western blot; (w), weak cross-reactive band; Weak, weakly positive in western blot.

## Discussion

In this study, we evaluated 65 epitope-specific human dystrophin antibodies in dog and mouse muscle (Table S1). To our knowledge, this is the first comprehensive analysis of a large collection of dystrophin antibodies in the murine and canine models of DMD. Among these antibodies, we identified 13 that recognized full-length dystrophin in both species (Figure 1, Table 1). We also found one antibody that reacted with canine but not murine dystrophin and two antibodies that reacted with murine but not canine dystrophin (Figures 2 and 3, Table 1). In addition, seven antibodies were assay-specific and they worked either on immunostaining or on western blot but not both (Figures 4 and 5, Table 1). One antibody appeared to have tissue specificity (Figure 6). It recognized dystrophin in the heart but not skeletal muscle (Figure 6, Table 1). Four antibodies did not react with mouse/dog dystrophin at all (Figure 7, Table 1). Interestingly, 11 antibodies detected an approximately 400 kD band in western blot of dystrophin-null muscles (Table 3). With the goal of applying our findings to preclinical study in the dog model, we selected 15 antibodies as the first line antibody for dog study (Table 4). These antibodies are highly specific in both immunostaining and western blot. We also identified another 15 antibodies that are suitable for dog muscle immunostaining but not ideal for western blot (Table 5).

Antibodies are among the most commonly used experimental reagents in basic and clinical research. Validating the specificity and selectivity of an antibody is thus of paramount importance (reviewed in [31], [32]). Historically, there have been many incidences in which a conclusion was drawn inappropriately due to the lack of sufficient knowledge on a particular antibody. For example, we recently discovered that dystrophin spectrin-like repeats 16 and 17 (R16/17) are essential for sarcolemmal localization of neuronal nitric oxide synthase (nNOS) [33], [34]. A panel of different antibodies and in situ nNOS enzyme activity assay were used to corroborate the finding. Prior to the publication of our study, Wang et al reported that nNOS was recruited to the sarcolemma by a micro-dystrophin gene that lacks R16/17 [35]. Unfortunately, the nNOS antibody used in the Wang et al study has not been fully validated in skeletal muscle.

Species-specific dystrophin antibodies offer a unique experimental advantage. For example, a human specific antibody can be used to confirm engraftment of human dystrophin in mice. In this regard, ’t Hoen et. al. have used human-specific MANDYS106 to characterize full-length human dystrophin transgenic mice [36]. Similarly, we have used human-specific Dys-3 to distinguish revertant fibers in mdx mice from myofibers that are transfected by human dystrophin [33]. In the current study, we discovered that in addition to their human dystrophin reactivity (www.glennmorris.org.uk/mabs.htm), MANEX4850D only reacted with dog dystrophin while MANEX1216B and MANEX45B only reacted with mouse dystrophin. The exact mechanism underlying the species-specific antibody recognition is not completely clear. However, it may very likely relate to amino acid differences in the epitope as demonstrated for MANDYS106 [17].

Assay-specific antibodies are frequently reported in the literature (reviewed in [31], [32]). In general, if an antibody only recognizes a denatured epitope, it usually works fine in western blot but poorly in immunostaining. On the other hand, if an antibody only reacts with the epitope in its native conformation, in general it tends to perform well in immunostaining but not western blot. It has been reported previously that MANDRA13, MANDRA14 and MANDYS141 worked poorly for immunostaining [22], [37], [38]. Our studies confirmed these results and suggest that these antibodies should be used for western blot only (Figure 5, Tables 1 and 2). We also found three antibodies (MANDYS111, MANDYS103 and MANDYS106) that did not work for western blot with dog or mouse muscle at all (Figure 4, Table 1), although they do work on western blots of human muscle (www.glennmorris.org.uk/mabs.htm). Apparently, these antibodies only recognized native dog and mouse dystrophin in frozen muscle sections and should be recommended for immunostaining only.

A surprising finding of this study is the discovery of a tissue-specific antibody MANHINGE4A (Figure 6, Table 1). This antibody preferentially recognized dystrophin in the heart but not skeletal muscle in both immunostaining and western blot (Figure 6). Tissue-specific reactivity has been described in an insulin receptor antibody [39]. Basically, that antibody is much more efficient when used to immunoprecipitate the insulin receptor from the placenta but not from erythrocytes [39]. Currently, we are not clear why MANHINGE4A only reacted with dystrophin in the heart. The epitope of this antibody is mapped at hinge 4, a region that links the long central rod domain to the cysteine-rich domain. At this point, we can only speculate that dystrophin hinge 4 may assume different conformations in the heart and skeletal muscle. Further studies are needed to elucidate the underlying mechanism(s).

As expected, we came across several antibodies that failed to recognize dystrophin at all under the conditions used in this study (Figure 7, Table 1). A likely explanation is that these antibodies are human-specific antibodies. In this regard, we have shown that Dys-3 only reacted with human dystrophin [33]. Additional studies are needed to fully characterize these antibodies.

On western blot, many antibodies not only revealed the full-length dystrophin protein (427 kD), but also lit up various smaller bands ranging from 100 to 400 kD (Table 2). There are several possible explanations for these cross-reactive bands. First, these smaller bands may represent proteolytic fragments of the full-length protein [11]. Second, they may be naturally occurring non-muscle isoforms of dystrophin such as Dp260, Dp140, Dp116 and Dp70 (reviewed in [9]). Third, we cannot completely exclude the possibility that these bands are actually not dystrophin at all [40]. They may have cross-reacted with other spectrin/actinin family proteins or yet unknown proteins.

With a subset of antibodies, we detected a near full size band (∼ 400 kD) in dystrophic muscles by some antibodies (Table 3). Based on our previous studies [37], [41], we suspect that this band might be utrophin. Utrophin is an autosomal homologue of dystrophin and it is up-regulated in dystrophic muscle (reviewed in [9]). Utrophin has a molecular weight of 395 kD. It shares a high degree of sequence homology with dystrophin although there are important functional differences between the two proteins [42]. In support of our reasoning, all the MANCHO antibodies were initially raised against sequence epitopes in human utrophin [37], [41]. Some of these antibodies were later found to react with human dystrophin [41]. In addition, several antibodies (such as MANDRA3) were raised against sequences that are identical in dystrophin and utrophin. Nevertheless, future studies are needed to confirm the identity of the band. The knowledge on dystrophin/utrophin cross-reactivity is extremely valuable for gene therapy study that is aimed at restoring dystrophin expression. Failure to validate the specificity of the antibody may result in erroneous judgment of gene transfer efficiency. On the other hand, we noted that none of the antibodies listed in Table 3 stained the sarcolemma in dystrophic dog muscle, although they do recognize up-regulated utrophin in the sarcolemma of DMD patient muscle [41].

We would like to point out that our study also has some limitations. First, to ensure a side-by-side comparison, we have used a standard protocol in our study. It is very likely that one may get a different result if experimental conditions/protocols are altered. Such changes may include (but not limited to) differences in embedding and fixation method, antibody dilution and muscle lysate preparation. Second, in this study, we only performed immunostaining and western blot. Additional studies are needed to characterize the usage in other applications (such as immunoprecipitation and enzyme-linked immunosorbent assay).

In summary, we have validated 65 dystrophin monoclonal antibodies for their use in the murine and canine DMD models. Our findings will serve as a benchmark to help investigators better determine the outcome of preclinical experimental therapies [21], [43].

## Supporting Information

Figure S1 Representative photomicrographs showing the definition of the signal intensity in immunostaining and western blot. **a,** Strong positive (+++) in immunostaining. **b,** Positive (++) in immunostaining. **c,** Weak positive (+) in immunostaining. **d,** Negative (−) in immunostaining. **e,** Positive (left lane) and weak positive (right lane) in western blot.
(TIF)

Table S1 A summary of all 65 antibodies examined in this study.
(XLSX)
