# Supplementary figures and images for: Characterization of 65 Epitope-Specific Dystrophin Monoclonal Antibodies in Canine and Murine Models of Duchenne Muscular Dystrophy by Immunostaining and Western Blot
Source: PLoS One. 2014 Feb 7;9(2):e88280. doi: 10.1371/journal.pone.0088280 (PMC3917863; doi:10.1371/journal.pone.0088280)

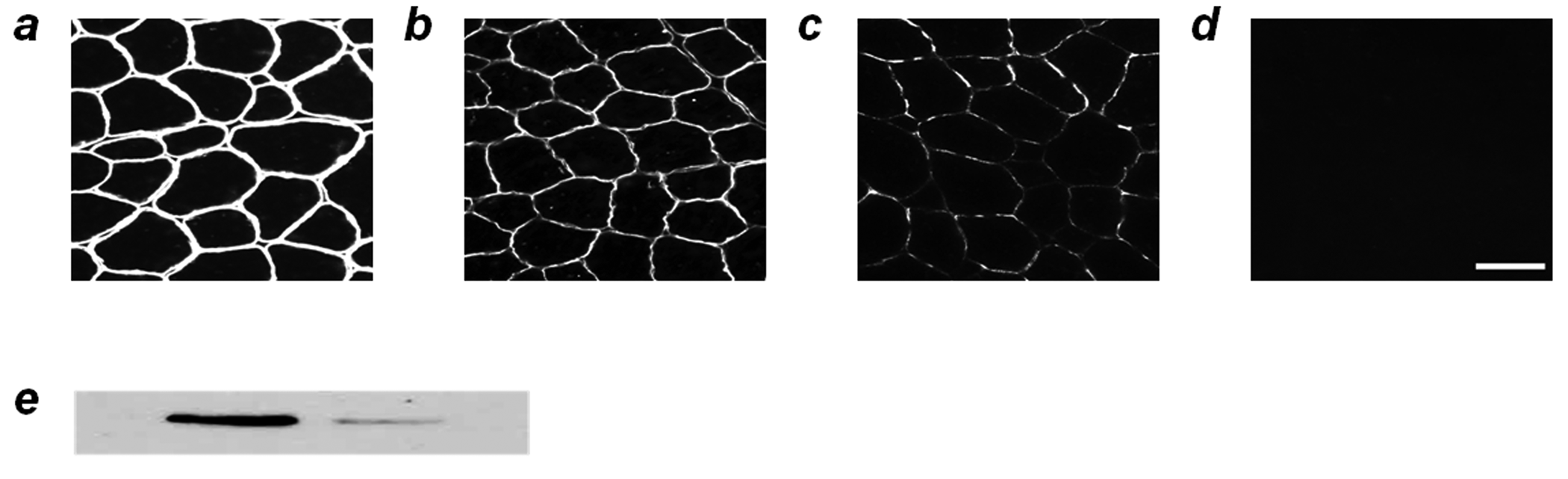

Supplement: Figure S1 — Representative photomicrographs showing the definition of the signal intensity in immunostaining and western blot. a, Strong positive (+++) in immunostaining. b, Positive (++) in immunostaining. c, Weak positive (+) in immunostaining. d, Negative (−) in immunostaining. e, Positive (left lane) and weak positive (right lane) in western blot. (TIF) [file pone.0088280.s001.tif]
